# Use of the bibliometric in rare diseases: taking Wilson disease personally

**DOI:** 10.1186/s13023-022-02459-7

**Published:** 2022-07-29

**Authors:** Lin Chen, Zhuoqi Lou, Yangxin Fang, Liya Pan, Jianhua Zhao, Yifan Zeng, Ying Wang, Nan Wang, Bing Ruan

**Affiliations:** 1 State Key Laboratory for Diagnosis and Treatment of Infectious Diseases, National Clinical Research Center for Infectious Diseases, Collaborative Innovation Center for Diagnosis and Treatment of Infectious Diseases, The First Affiliated Hospital, Zhejiang University School of Medicine, No. 79 Qingchun RoadZhejiang Province, Hangzhou, 310003 China; 2 College of Polytechnic Institute, Zhejiang University, No. 38 Zheda Road, Hangzhou, 310027 Zhejiang China; 3 The Affiliated Hospital of Stomatology, School of Stomatology, Zhejiang University School of Medicine, and Key Laboratory of Oral Biomedical Research of Zhejiang Province, No. 395, Yan’an Road, Hangzhou, 310006 Zhejiang China; 4 Department of Geriatric Endocrinology, The First Affiliated Hospital, Anhui Medical University, No. 218 Jixi Road, Hefei, 230022 Anhui China

**Keywords:** Academic productivity, Wilson disease, Bibliography, Bibliometric

## Abstract

**Background:**

Bibliometric have been widely applied to the evaluation of academic productivity. However, those of individuals or institutions on a specific disease have not been explored. The aim of the present study is to conduct a bibliometric analysis of particular rare disease and investigate whether those doctors and hospitals with higher index screened by this method specialize in this disease.

**Methods:**

A representative rare disease, Wilson disease (WD), was searched on Clarivate Analytics’ Web of Science and Elsevier’s Scopus, which was published in English between 1 January 2001 and 31 December 2020. Clinical authors and medical institutions with the most papers were screened, and their total number of publications and citations, h-index and g-index were computed and then ranked by h-index.

**Results:**

A total of 6856 and 6193 papers and 200 and 160 authors were got from WoS and Scopus, respectively. Scopus provided 160 institutions. The above bibliometric indices were calculated in 100 researchers and 80 institutions, and top 30 authors (Top-30a) and top 20 institutions (Top-20i) of them based on the h-index were listed in the tables. Top-30a came from seven specialties and 13 countries whose median (interquartile range) h-index was 14 (12–19.5) (range 10–28) which was located between associate and full professors in some other disciplines. Top-20i was distributed in 13 countries whose mean ± standard deviation of the h-index was 15 ± 4.9 (range 10–27).

**Conclusions:**

The related specialists and medical institutions of WD screened by specific disease bibliometric analysis are eminent and credible and benefit WD patients to obtain reliable medical treatment. This model may be suitable for other rare diseases.

**Supplementary Information:**

The online version contains supplementary material available at 10.1186/s13023-022-02459-7.

## Background

In the field of academic medicine, scientific productivity is still a crucial and more objective factor for evaluating scholarly achievements and promotion [1, 2]. Bibliometric provides a quantitative method that is widely accepted thus far. The h-index [3] and g-index [4] are two of the most broadly known and used bibliometric parameters on the individual and departmental levels [5–9]. “The h-index is defined as an individual having h papers with at least h citations” [3]. It balances both the total publications and citations per publication, and has good stability since it is less affected by low-cited articles [10]. The g-index means that the most cited g articles earned at least g^2^ citations [4]. It could provide weight to a h-index that is too-low when a small number of very influential papers result in an uneven distribution of citations.

At present, bibliometric studies focus on the global academic productivity of individuals or departments, rather than a single disease. It is impossible for an expert to have high scholarly achievement in varied diseases. A single disease’s bibliometric analysis may be necessary when we need to assess the influence of a doctor on a particular disease, especially for the diagnosis and therapy of rare diseases.

To date, there is no exact and uniform definition and count of rare diseases. A disease that affects fewer than 200,000 people in the United States is rare [11]. Meanwhile, in the European Union in 2000, less than 5 in 10,000 people was considered a rare disease [12]. It is estimated the number of rare diseases is more than 10,000 [13]. Therefore, all rare diseases face a similar clinical dilemma, since most clinicians and departments do not have more opportunities to deal with rare disease patients which precisely prevents them from accumulating adequate experience to recognize and manage rare diseases. Then, diagnosis is often delayed or wrong followed by increased financial burden and physical torment. Therefore, the answers to the following questions may be particularly critical: Who are authentic clinical specialists of rare diseases? How can their clinical competence be evaluated? Would rare disease bibliometric research for individuals and clinical institutions be the perfect solution?

Wilson disease (WD) is a rare but potentially treatable and inherited disorder of copper metabolism with a prevalence of 2.9–5.87 per 100,000 [14–17]. As far as I know, there is abundant literature on WD available for analysis. In this study, we took WD as an example of a rare disease to conduct a bibliometric analysis based on literature published from 2001 to 2020, investigated the academic productivity of experts and institutions in this special field, and explored whether the experts and institutions singled out by particular disease bibliometrics were reliable in the field of that rare disease.

## Methods

### Search and inclusion criteria

All publications were searched using “Hepatolenticular Degeneration” as the medical subject heading (MeSH) term and related entry terms which came from the National Center for Biotechnology Information (NCBI). The above terms were retrieved in the “Article title, Abstract and Keywords” of Elsevier’s Scopus and the “Topic” of Clarivate Analytics’ Web of Science (WoS) to analyze the bibliometric information of relevant dissertations that were published between 1 January 2001 and 31 December 2020 in English and document types were limited to articles or review articles or case report or letters or clinical trial or report (Additional file 1). The study data were collected in December 2021 over one week period. For the purpose of this study, the inclusion criteria of this literature were that (1) the details must be related to WD (known or called also hepatolenticular degeneration); and (2) their authors need to involve clinicians, not just fundamental science (nonclinical) researchers. We can only identify whether the author is a clinical researcher based on the institution of the latest published articles, and those obscure and uncertain results would not be involved in this study.

### Citation data sources

The list of authors with the most papers and their publication data were extracted from Scopus and WoS databases on the same day. One author may have various abbreviations of the first name, for instance, Michael L Schilsky, Michael Schilsky, MICHAEL Schilsky, ML Schilsky, M L Schilsky and M Schilsky are the same person. We sorted by author's last name to maximize the discovery of all his articles. Article title, authors, department, institution, abstract, PubMed unique identifier, publication year and cited times were extracted. Our colleagues checked and merged raw data from two databases to unify the author’s name and ignore the nonclinical researcher and erase the duplicate items and unrelated themes. The top cited articles and the list of institutions were provided by Scopus. Their publication data were screened as above. Suspicious items were identified from the original literature. The above data were then saved as a spreadsheet for bibliometric calculations. We cannot identify the most-paper authors’ sex, age, and work situation, for instance, in-service, retired or emeritus. Although we tried our best to retrieve the initials of those authors in various forms, the omission of a few papers may still be inevitable. Since there is no uniform named standard and some institutions have renamed, merged or ceased to exist, we did not consider the literature data of institutions sourced from Scopus to be accurate.

### Bibliometric indices

The most-paper authors’ following indices were calculated: total number of publications; the total number of citations; h-index; g-index. The top 20 most-cited papers (Top-20p) and their authors were listed. We ranked the top 30 authors/doctors (Top-30a) worldwide according to their scientific research output and h-index. The network mapping of Top-30a and their coauthors was charted with the VOSviewer program [18], which reflects their influence power in this specialty field. The h-index, which is used to calculate individual academic output, [3] was borrowed here to calculate the academic performance of organizations and obtain the top 20 medical institutions (Top-20i).

## Results

### Characteristics of publications

A total of 6856 and 6193 papers related to WD were searched from WoS and Scopus respectively. The different names of article types in these databases resulted in discrepancies in the results, although the MeSH was the same. The Top-20p, which was taken part in by clinicians, is shown in Table 1. Seven of the top 10 most-cited papers are guidelines or reviews, and most of them were published 10 years ago [19–25]. Notably, compared with only one laboratory study on the mouse model of WD [26], the other 19 studies were all clinical investigations or reviews. The highest citation, 772, was produced by a practice guideline article [23], which belongs to Eve A. Roberts and Michael L. Schilsky, published in Hepatology in 2008 and represented the position of the American Association for Study of Live Disease (AASLD). Michael L. Schilsky and Peter Ferenci, two prestigious professors, were the principal coauthors in Top-20p. Michael L. Schilsky [27], professor of medicine and medical director in Adult Liver Transplant at Yale-New Haven Transplantation Center, was involved in seven publications, four of which ranked first to fourth [19, 22–24] on Top-20p. Moreover, the first and fourth papers are clinical practice guidelines of AASLD and the European Association for the Study of the Liver (EASL) respectively. Another eminent researcher, Peter Ferenci [28, 29], liver expert in the division of gastroenterology and hepatology, comes from the department of medicine III of Medical University Vienna. He was the chairman of EASL Clinical Practice Guidelines: Wilson’s disease [24] and had 6 publications listed on Top-20p.

**Table 1:** Top 20 most-cited papers related to Wilson Disease from 2001 to 2020, whose authors involved clinicians

Rank	Title	Total cites	Authors	Journal	Year	Type
1	Diagnosis and treatment of Wilson disease: An update	772	Eve A. Roberts, Michael L. Schilsky	Hepatology	2008	Guideline
2	Wilson's disease	745	Aftab Ala, Ann P. Walker, Keyoumars Ashkan, James S. Dooley, Michael L. Schilsky	Lancet	2007	Review
3	Diagnosis and phenotypic classification of Wilson disease	549	Peter Ferenci, Karel Caca, Georgios Loudianos, Giorgina Mieli-Vergani, Stuart Tanner, Irmin Sternlieb, Michael L. Schilsky, et al	Liver International	2003	Review
4	EASL Clinical Practice Guidelines: Wilson's disease	548	Peter Ferenci, Anna Czlonkowska, Wolfgang Stremmel, Roderick Houwen, William Rosenberg, Michael L. Schilsky, et al	Journal of Hepatology	2012	Guideline
5	Wilson's disease and other neurological copper disorders	345	Oliver Bandmann, Karl Heinz Weiss, Stephen Kaler	The Lancet Neurology	2015	Review
6	Clinical presentation, diagnosis and long-term outcome of Wilson's disease: A cohort study	337	UTA Merle, Matthias R. Schaefer, Peter Ferenci, Wolfgang Stremmel	Gut	2007	Article
7	A practice guideline on Wilson disease	307	Eve A. Roberts, Michael L. Schilsky	Hepatology	2003	Review
8	Wilson Disease	266	Jonathan D Gitlin	Gastroenterology	2003	Review
9	Wilson's disease in children: 37-year experience and revised King's for liver transplantation	230	Anil Dhawan, Rachel M. Taylor, Paul Cheeseman, Pamela De Silva, Leah Katsiyiannakis, Giorgina Mieli-Vergani	Liver Transplantation	2005	Article
10	Treatment of Wilson disease with ammonium tetrathiomolybdate—IV. Comparison of tetrathiomolybdate and trientine in a double-blind study of treatment of the neurologic presentation of Wilson disease	199	George J. Brewer, Fred Askari, Matthew T. Lorincz, Martha Carlson, Michael L. Schilsky, Karen J Kluin, et al	Archives of Neurology	2006	Article
11	Wilson's disease: An update	197	Shyamal K. Das, Kunal Ray	Nature Clinical Practice Neurology	2006	Review
12	A genetic study of Wilson's disease in the United Kingdom	186	Alison J Coffey, Miranda Durkie, Stephen Hague, Kirsten McLay, Jennifer Emmerson, Christine Lo, Stefanie Klaffke, Christopher J Joyce, Anil Dhawan, et al	Brain	2013	Article
13	Regional distribution of mutations of the ATP7B gene in patients with Wilson disease: Impact on genetic testing	177	Peter Ferenci	Human Genetics	2006	Review
14	High copper selectively alters lipid metabolism and cell cycle machinery in the mouse model of Wilson disease	159	Dominik Huster, Tina D. Purnat, Jason L. Burkhead, Martina Ralle, Oliver Fiehn, Franziska Stuckert, N Erik Olson, Daniel Teupser, Svetlana Lutsenko	Journal of Biological Chemistry	2007	Article
15	Wilson disease: Description of 282 patients evaluated over 3 decades	157	Arun B. Taly, Meenakshi-Sundaram S., Sanjib Sinha, HS. Swamy, GR. Arunodaya	Medicine	2007	Article
16	Late-Onset Wilson's Disease	155	Peter Ferenci, Anna Czlonkowska, UTA Merle, Szalay Ferenc, Grazyna Gromadzka, Cihan Yurdaydin, Wolfgang Vogel, Radan Bruha, Hartmut T Schmidt, Wolfgang Stremmel	Gastroenterology	2007	Article
17	Neurologic Wilson's disease	148	Matthew T. Lorincz	Annals of the New York Academy of Sciences	2010	Review
18	Wilson's disease: Cranial MRI observations and clinical correlation	144	Sanjib Sinha, Arun B. Taly, Saiprasad Ravishankar, et al	Neuroradiology	2006	Article
19	Screening for Wilson disease in acute liver failure: A comparison of currently available diagnostic tests	142	Jessica D. Korman, Irene Volenberg, Jody Balko, Joe Webster, Frank V Schiodt, Robert H. Squires Jr, Robert J Fontana, William M. Lee, Michael L. Schilsky,	Hepatology	2008	Article
20	Diagnostic value of quantitative hepatic copper determination in patients with Wilson's disease	141	Peter Ferenci, Petra Steindl-Munda, Wolfgang Vogel, Wolfgang Jessner, Michael Gschwantler, Rudolf Stauber, et al	Clinical Gastroenterology and Hepatology	2005	Article

### Top authors’ academic productivity

**Fig. 1:**
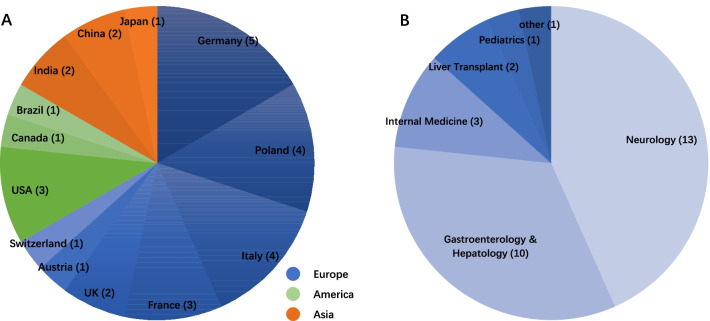
Geographical (**A**) and departmental (**B**) distribution of top 30 authors

**Fig. 2:**
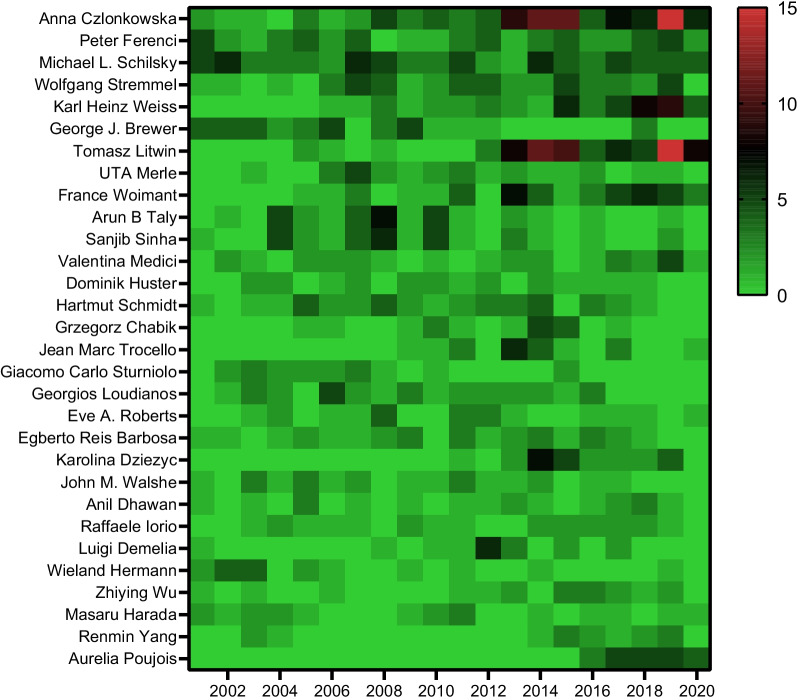
Heatmap of the top 30 authors’ number of active years of publication

WoS and Scopus presented 200 and 160 authors whose median (interquartile range, IQR) paper numbers were 25(19, 35) (range 13–102) and 12(10, 17) (range 9–75) respectively (data not shown). The top 100 authors on WoS with the most articles were retrieved, whose article numbers ranged from 18 to 102. Then, their records on Scopus were downloaded and merged with those of WoS to calculate the h-index. According to the h-index, Top-30a who were productive in the WD field, coming from seven specialties and 13 countries (Fig. 1A), are listed in Table 2. The median (IQR) of the h-index is 14 (12–19.5), ranging from 28 to 10. Anna Czlonkowska tops the list with the highest h-index, 28, and the largest number of papers, 98, which generated 3039 citations. Peter Ferenci and Michael L. Schilsky followed behind closely with h-index values of 27 and 26, respectively. Nevertheless, their g-indices were higher than Anna Czlonkowska’s and occupied the top two positions. Tomasz Litwin, similar to his colleague Anna Czlonkowska, had a number of articles and a disproportionate h-index. In terms of total cites, Michael L. Schilsky, who was coauthor of both AASLD and EASL’s guidelines about WD [23, 24], ranked first by virtue of 4953 citations of 77 articles. Furthermore, his g-index score, 70, was far higher than his peers, just like his total cites. Surprisingly, Dominik Huster and Eve A. Roberts, ranking 13th and 19th based on the h-index, merely relied on 20 papers to earn 1276 citations and 1504 citations, respectively. We made a heatmap based on the number of articles published by each author per year (Fig. 2). It showed that the academic output of top researchers, such as Anna Czlonkowska, Peter Ferenci, Michael L. Schilsky and Wolfgang Stremmel, was not only consistent but also productive. Impressively, Jean Marc Trocello and Aurelia Poujois, who both came from the French National Reference Centre for Wilson Disease (Paris), and Karolina Dziezyc (Warsaw) have had a good start to the last decade and may have a bright future (Fig. 2).

**Table 2:** Top 30 authors who are clinicians and had the highest h-index according to their articles related to Wilson Disease from 2001 to 2020

Rank	Name	h-index	g-index	Total articles	Total cites	Specialty	Department & Institution
1	Anna Czlonkowska	28	51	98	3039	Neurology	Second Department of Neurology, Institute of Psychiatry and Neurology, Warsaw, Poland
2	Peter Ferenci	27	53	53	3726	Gastroenterology and Hepatology	Division of Gastroenterology and Hepatology, Medical University of Vienna, Vienna, Austria
3	Michael L. Schilsky	26	70	77	4953	Liver Transplant	Departments of Medicine and Surgery, Yale University Medical Center, New Haven, CT, USA
4	Wolfgang Stremmel	25	48	48	2556	Gastroenterology and Hepatology	Department of Gastroenterology and Hepatology, University Hospital of Heidelberg, Heidelberg, Germany
5	Karl Heinz Weiss	23	40	51	1674	Gastroenterology and Hepatology	Department of Gastroenterology and Hepatology, University Hospital of Heidelberg, Heidelberg, Germany
6	George J. Brewer	23	36	36	1801	Internal Medicine	Department of Human Genetics and Department of Internal Medicine, University of Michigan Medical School, Ann Arbor, USA
7	Tomasz Litwin	21	38	74	1695	Neurology	Second Department of Neurology, Institute of Psychiatry and Neurology, Warsaw, Poland
8	France Woimant	19	27	45	846	Neurology	Neurology Department and National Reference Centre for Wilson's Disease, AP-HP, Lariboisière University Hospital, Paris, France
9	UTA Merle	19	26	26	1318	Gastroenterology and Hepatology	Department of Gastroenterology, University Hospital Heidelberg, Heidelberg, Germany
10	Arun B Taly	18	32	33	1049	Neurology	Department of Neurology, National Institute of Mental Health and Neuro Sciences, Bangalore, India
11	Sanjib Sinha	17	31	33	1004	Neurology	Department of Neurology, National Institute of Mental Health and Neuro Sciences, Bangalore, India
12	Valentina Medici	17	29	29	918	Gastroenterology and Hepatology	Division of Gastroenterology and Hepatology, Department of Internal Medicine, University of California Davis, Sacramento, USA
13	Dominik Huster	17	20	20	1276	Gastroenterology and Oncology	Department of Gastroenterology and Oncology, Deaconess Hospital Leipzig, Academic Teaching Hospital University of Leipzig, Germany
14	Hartmut Schmidt	17	31	36	1013	Transplantation	University Hospital of Muenster, Muenster, Germany
15	Grzegorz Chabik	14	18	18	602	Neurology	Second Department of Neurology, Institute of Psychiatry and Neurology, Warsaw, Poland
16	Jean Marc Trocello	14	20	20	566	Neurology	Neurology Department and National Reference Centre for Wilson's Disease, AP-HP, Lariboisière University Hospital, Paris, France
17	Giacomo Carlo Sturniolo	14	18	18	631	Gastroenterology	Department Surgery & Gastroenterol, University of Padova, Padova, Italy
18	Georgios Loudianos	13	30	30	1032	NA	Ospedale Regionale Microcitemie, Cagliari, Italy
19	Eve A. Roberts	13	20	20	1504	Gastroenterology	Division of Gastroenterology, Hepatology and Nutrition, The Hospital for Sick Children, University of Toronto, Canada,
20	Egberto Reis Barbosa	13	23	28	570	Neurology	Department of Neurology, University of São Paulo Medical School, São Paulo, Brazil
21	Karolina Dziezyc	13	21	25	470	Neurology	Second Department of Neurology, Institute of Psychiatry and Neurology, Warsaw, Poland
22	John M. Walshe	13	22	22	541	Internal Medicine	Formerly of Addenbrookes Hospital, Cambridge and the Middlesex Hospital, London, UK
23	Anil Dhawan	12	19	19	858	Paediatric Hepatology	Paediatric Liver, GI and Nutrition Centre and Mowat Labs, King's College Hospital, Denmark Hill, London, UK
24	Raffaele Iorio	12	21	21	576	Pediatrics	Department of Pediatrics, University of Naples Federico II, Naples, Italy
25	Luigi Demelia	12	16	17	287	Gastroenterology	Department of Gastroenterology, Hospital of University of Cagliari, Cagliari, Italy
26	Wieland Hermann	11	17	17	435	Neurology	Department of Neurology, SRO AG Spital Langenthal, Langenthal, Switzerland
27	Zhiying Wu	11	18	18	449	Neurology	Department of Neurology and Research Center of Neurology in Second Affiliated Hospital, Zhejiang University School of Medicine, Hangzhou, China
28	Masaru Harada	10	18	19	327	Internal Medicine	Third Department of Internal Medicine, School of Medicine, University of Occupational and Environmental Health, Fukuoka, Japan
29	Renmin Yang	10	15	15	247	Neurology	Department of Neurology, The Affiliated Hospital of the Neurology Institute, Anhui University of Chinese Medicine, Hefei, China
30	Aurelia Poujois	10	15	22	245	Neurology	Neurology Department and National Reference Centre for Wilson's Disease, AP-HP, Lariboisière University Hospital, Paris, France
Median (range)	14 (10–28)	22.5 (15–70)	25.5 (15–98)	888 (245–4953)			
Inter-quartile range	12–19.5	18–33	19–38.3	523.3–1546.5			

*USA* United States of American, *AP-HP* Public Assistance-Paris Hospitals, *UK* United Kingdom

Broadly speaking, Asian researchers’ academic productivity, especially Japan and China, at least in English publications, was inefficient. The higher h-index, g-index, total articles and total cites of authors, by contrast, all belonged to Europe and the United States (Table 2, Fig. 1A). In addition, approximately half of Top-30a came from neurology and one third of them were gastroenterologists and hepatologists (Fig. 1B). Most of Top-30a were employees of the hospital affiliated with the medical university (Table 2).

**Fig. 3:**
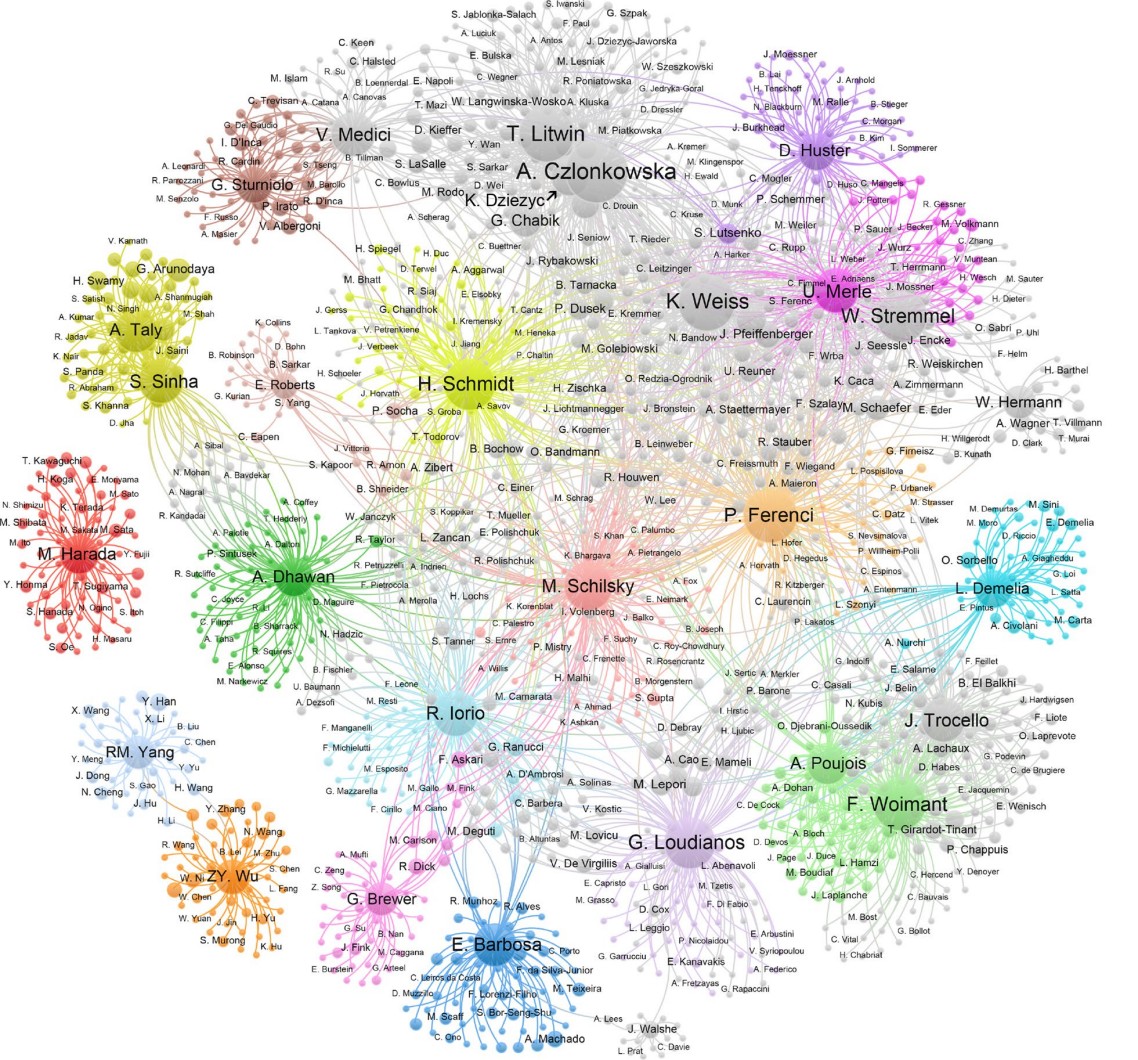
Network visualization of the top 30 authors and their coauthors using VOSviewer. The line indicates that two people have cooperation, and the size of the circle demonstrates relative times they collaborate with others

The network relationships of Top-30a and their coauthors are presented in Fig. 3. Obviously, in the lower left corner, Chinese researchers, RM. Yang, and ZY. Wu, and Japanese scholar, M. Harada, do not have academic cooperation with the right sophisticated and interlinked cluster, which was made up of European and American peers. In line with the performance of Top-30a in Table 2, generally, the higher up authors also had larger circles, more complex network relationships and a more central position in Fig. 2, for instance, A. Czlonkowska, P. Ferenci, K. Weiss and M. Schilsky. Top-30a in the same area and with the same color generally belong to the same cluster or even the same institution.

### Top institution’ academic productivity

Scopus demonstrated 160 institutions based on article counts whose median (IQR) was 25 (19, 35) (range 16–88). The top 80 (50%) institutions’ papers, which ranged from 25 to 88, were proven and their related indices were calculated. In light of the h-index again, Table 3 ranks the Top-20i located in 13 countries whose h-index was between 10 and 27, with a mean ± standard deviation of 15 ± 4.9. In contrast with the affiliated institutions of Top-30a in Table 2, unexpectedly, all indices of Top-20i were much lower, which was why we only retrieved 20. Due to universal nomenclature being nonexistent and job-hopping, the above differences may be reasonable. The Institute of Psychiatry and Neurology in Warsaw, Poland, whose four authors are presented in the Top-30a list (Table 2), ranked first in the Top-20i list with 27 of h-index, 45 of g-index and 2440 total cites yielded by 88 papers, followed by the University Hospital of Heidelberg, from which three researchers in the Top-30a list came (Tables 2, 3). In addition, there were four institutions that acquired very higher cites that rely on relatively fewer articles: University (Hospital) of California Davis, First Faculty of Medicine of Charles University, King's College of Hospital and University of Toronto Hospital for Sick Children. Geographically, as with Top-30a, Germany, the US and Italy remained the main players. Furthermore, Switzerland, Japan and China disappeared from Table 3, albeit they had outstanding individual performance (Table 2).

**Table 3:** Top 20 institutions contributing manuscripts of Wilson Disease between 2001–2020

Rank	Institution	Location	h-index	g-index	Total articles	Total Cites
1	Institute of Psychiatry and Neurology	Warsaw, Poland	27	45	88	2440
2	University Hospital of Heidelberg	Heidelberg, Germany	25	43	54	2274
3	National Institute of Mental Health and Neuro Sciences	Bangalore, India	20	35	39	1276
4	University (Hospital) of Leipzig	Leipzig, Germany	19	27	27	1172
5	University of Michigan Medical School (Hospital)	Ann Arbor, USA	19	25	25	1776
6	Medical University (Hospital) of Vienna	Vienna, Austria	18	31	31	1717
7	AP-HP, Lariboisière University Hospital	Paris, France	18	29	40	856
8	University (Hospital) of Naples Federico II	Naples, Italy	15	26	26	856
9	University (Hospital) of California Davis	California, USA	15	22	22	1669
10	University (Hospital) of Padova	Padova, Italy	15	18	18	640
11	Münster University Hospital	Münster, Germany	14	26	26	739
12	University of São Paulo School of Medicine (Hospital)	São Paulo, Brazil	13	23	29	581
13	First Faculty of Medicine, Charles University	Prague, Czech Republic	13	19	19	1181
14	Sanjay Gandhi Post Graduate Institute (Hospital) of Medical Sciences	Lucknow, India	12	18	20	337
15	University Medical Center Utrecht	Utrecht, Netherlands	12	15	15	855
16	University (Hospital) of Cagliari	Cagliari, Italy	11	22	24	494
17	King's College of Hospital	London, UK	10	19	19	1348
18	University of Toronto Hospital for Sick Children	Toronto, Canada	10	16	16	1232
19	Yale University School of Medicine (Hospital)	New Haven, USA	10	15	15	379
20	Asan Medical Center, University of Ulsan College of Medicine	Seoul, South Korea	10	15	15	294
Median ± Standard deviation (range)			15.3 ± 4.9 (10–27)	24.5 ± 8.7 (15–45)	28.4 ± 17.2 (15–88)	1105.8 ± 622.6 (294–2440)

*USA* United States of American, *AP-HP* Public Assistance-Paris Hospitals, *UK* United Kingdom

## Discussion

Taking the WD as an example and using bibliometric methods, we have screened the Top-30a and Top-20i, which may be the most trustworthy in the diagnosis and treatment of WD. This model may provide a basis to help WD patients choose the appropriate doctor or medical institution and may even be beneficial for undiagnosed dubious patients.

Regarding bibliometric, the h-index is the most broadly used and accepted measure of scholarly productivity and even for hiring, promotion, award and funding decisions [1, 6, 30–32]. Some studies have been conducted in fields such as neurosurgery, pediatric, academic otolaryngology, anesthesia, radiology and chronic pain medicine [2, 5–9]. This study may be the first attempt to calculate the h-index using papers from a specific disease rather than all literature. The h-index increases gradually with advancing academic ranks from lecturer to assistant professor, associate professor (AP), full professor (FP) and finally chairman [32]. Hirsch found that a general value for promotion to AP or FP would be ~ 12 and ~ 18 respectively [3]. The h-index is robust because of its insensitivity to lowly cited articles in a researcher’s album [5, 10, 32]. The common view is that the h-index will never fall and with no doubt is influenced by the author’s scholarly career period, which is seen as a drawback. However, this could be a benefit in the rare disease field, where senior physicians tend to have abundant experience.

Almost all physicians of Top-30a are affiliated with university hospitals or research institutes (Table 2). Top-20i better illustrates this point (Table 3). Based on data from 2001 to 2020 alone and limited in particular disease (WD), the median (IQR) h-index of Top-30a is 14 (12–19.5), nearly equal to that of FP of chronic pain physicians in the USA: 16.5 (6, 30) [2], lower than that of anesthesia FP in the UK: 21 (16–26), both in their whole period [7]. Compared to the general academic pediatrician, the median h-index (14) and g-index (22.5) of Top-30a were slightly lower than those of FP (16 and 29) and markedly higher than AP (6 and 11) [9]. Although we did not list researchers’ titles, by comparison with the h- and g-index of AP and FP of other disciplines, we believe that these data obtained from our method can prove to us that these experts’ and institutions’ academic productivity in the field of WD is sufficiently convincing, they are also most likely to be good at WD. In fact, considering the enormous workload, we cannot retrieve all researchers and institutions listed by WoS and Scopus. We believe that the researchers/institutions who appear on this list are credible. Furthermore, there are four departments, coming from Switzerland, China and Japan, which appeared on the Top-30a table (Table 2) but disappeared on the Top-20i table (Table 3). We suspect that job-hopping had spread their work across different organizations, which may be the cause of the above results.

The European, American and Indian outputs were distinctly better than those of the rest of Asia, such as Japan and China (Tables 2 and 3, Fig. 1A). Language family [33] and collaboration may be the inescapable reasons (Fig. 3). Figure 3 demonstrates that Japanese and Chinese researchers had no academic collaboration with other clusters. Since bibliometric indices do not consider author rank in the manuscripts, multiple coauthors receiving equal credit, the citation and h-index can be strongly influenced by the size of his circle of collaborators [32]. The present study did not involve non-English papers; if these nonnative English speakers’ academic publications in their home countries were included, especially Japan and China, the rankings might change dramatically. We must always clearly realize that these lists only display academic productivity on WD field published in English between 2001 and 2020, which does not mean that the physicians on the list are definitely better at other periods than other scholars in other disease fields. If this method is applied to a specific country, such as Brazil, Japan, China and other non-English speaking countries, it may be more practical to use their native language papers as the object of analysis.

Although, academic influence about some disease does not completely equate to clinical diagnosis and treatment competence. In the domain of rare diseases with the background of rare patients, we believe that those who can sustain consistent and productive scholarly output can be relied upon by these patients. Therefore, the above bibliometric results are dependable. For rare disease patients, it could be used as a clue to help them discover the most suitable doctors and specialist institutions, which are bound to help reduce misdiagnosis and mistherapy and reduce the disease burden on individuals and society. Rare disease researchers will have access to more rare clinical cases and experience, which will be more beneficial to their scientific study. An ideal format, we conceive, might be an application that can be installed and operated on smartphones and computers. When the user enters doubtful or definitive diagnosis keywords in the search box, he will obtain relevant helpful experts and medical institutions.

Some limitations might influence the reliability of our results. First, we cannot include all authors and institutions listed by WoS and Scopus, which may omit some outstanding targets. However, from the perspective of our research purposes, we must ensure that the doctors on the list are excellent, and comprehensiveness is not compulsory. Their median h-index is 14 which is already higher than the AP of many disciplines and nearly reaches the level of FP. Therefore, their academic power is believable. Comparing the paper number of 200 authors (range 13–102) listed on WoS and the top 100 writers of them with the most publications (range 18–102) that were retrieved by us, we hold the opinion that the comprehensiveness is acceptable. The Top-20i also had the same situation. Second, the confidence level of our results only is proven by comparing their bibliometric indices with other subjects and specialties, whereas there is no dependable external validation. Peer review is probably a common practice; however, we doubt that they cannot ignore the impact of academic productivity. Third, WD is a sufficiently researched rare disease, and its related articles are abundant. When we handle other rare diseases, for instance, Dubin-Johnson Syndrome, which only has 3690 publications searched as above on WoS (data not shown) and may also include many unrelated topics, the situation could be entirely different, and the results could be obscure. Searching in larger categories may be a solution, such as inherited jaundice or inherited liver disease. Further research is still needed to verify these findings.

## Conclusion

As the research has demonstrated, through specific disease bibliometric analysis, we calculated a number of academic productivity indices of researchers and medical institutions in the field of WD. According to the h-index, we ranked and screened out the relevant credible specialists and specialized medical institutions that benefit WD patients to obtain most appropriate medical treatment. This model may be applied to other rare diseases and perhaps to some intractable diseases.

## Supplementary Information

**Additional file 1.** The search strategy of database.

## Data Availability

The data that support the findings of this study are available from the corresponding author upon reasonable request.

## Abbreviations

AASLD: American Association for Study of Live Disease
AP: Associate professor
EASL: European Association for the Study of the Liver
FP: Full professor
IQR: Interquartile range
MeSH: Medical subject headings
NCBI: National Center for Biotechnology Information
Top-30a: Top 30 authors
Top-20i: Top 20 medical institutions
Top-20p: Top 20 most-cited papers
WD: Wilson disease
WoS: Web of Science
