# Supplementary material for: Use of the bibliometric in rare diseases: taking Wilson disease personally
Source: Orphanet J Rare Dis. 2022 Jul 29;17:297. doi: 10.1186/s13023-022-02459-7 (PMC9335981; doi:10.1186/s13023-022-02459-7)
Supplement: Supplementary file 1 — Additional file 1. The search strategy of database. [file 13023_2022_2459_MOESM1_ESM.docx]

**Search strategy in Web of Science:**

((((((((((((((((((((((((((((((((((((((((((((((((TS=(hepatolenticular degeneration)) OR TS=(Degeneration, Hepatolenticular)) OR TS=(pneumosclerosis)) OR TS=(Wilson Disease)) OR TS=(Wilson's Disease)) OR TS=(wilson Disease)) OR TS=(Cerebral pneumosclerosis)) OR TS=(Cerebral pseudosclerosis)) OR TS=(pseudosclerosis, Cerebral)) OR TS=(pneumosclerosis, Cerebral)) OR TS=(Hepatolenticular Degeneration Syndrome)) OR TS=(Degeneration Syndrome, Hepatolenticular)) OR TS=(Degeneration Syndromes, Hepatolenticular)) OR TS=(Hepatolenticular Degeneration Syndromes)) OR TS=(Syndrome, Hepatolenticular Degeneration)) OR TS=(Syndromes, Hepatolenticular Degeneration)) OR TS=(Hepato-Neurologic Wilson Disease)) OR TS=(Diseases, Hepato-Neurologic Wilson)) OR TS=(Hepato Neurologic Wilson Disease)) OR TS=(Hepato-Neurologic Wilson Diseases)) OR TS=(Wilson Disease, Hepato-Neurologic)) OR TS=(Wilson Diseases, Hepato-Neurologic)) OR TS=(Hepatocerebral Degeneration)) OR TS=(Degeneration, Hepatocerebral)) OR TS=(Degenerations, Hepatocerebral)) OR TS=(Hepatocerebral Degenerations)) OR TS=(kindier-Wilson Disease)) OR TS=(Diseases, kindier-Wilson)) OR TS=(kindier Wilson Disease)) OR TS=(kindier-Wilson Diseases)) OR TS=(westphalt-strumpel Syndrome)) OR TS=(westphalt strumpel Syndrome)) OR TS=(westphalt-strumpel Syndromes)) OR TS=(Copper Storage Disease)) OR TS=(Copper Storage Diseases)) OR TS=(Disease, Copper Storage)) OR TS=(Diseases, Copper Storage)) OR TS=(Storage Disease, Copper)) OR TS=(Storage Diseases, Copper)) OR TS=(Progressive Lenticular Degeneration)) OR TS=(Degeneration, Progressive Lenticular)) OR TS=(Lenticular Degeneration, Progressive)) OR TS=(neurohematic Degeneration)) OR TS=(Degeneration, neurohematic)) OR TS=(Degenerations, neurohematic)) OR TS=(neurohematic Degenerations)) OR TS=(Hepatic Form of Wilson Disease)) OR TS=(Wilson Disease, Hepatic Form))

Document Types: Articles or Review Articles or Case Report or Letters or Clinical Trial or Report

Languages: English

Datebase: Web of Science Core Collection or BIOSIS Citation Index or MEDLINE or FSTA- the food science resource or SciELO Citation Index

Publication date: 2001-01-01 to 2020-12-31

**Search strategy in Scopus:**

( TITLE-ABS-KEY ( hepatolenticular AND degeneration ) OR TITLE-ABS-KEY ( degeneration, AND hepatolenticular ) OR TITLE-ABS-KEY ( pseudosclerosis ) OR TITLE-ABS-KEY ( wilson AND disease ) OR TITLE-ABS-KEY ( wilson's AND disease ) OR TITLE-ABS-KEY ( wilsons AND disease ) OR TITLE-ABS-KEY (Cerebral AND Pseudosclerosis) OR TITLE-ABS-KEY (Cerebral AND Pseudoscleroses) OR TITLE-ABS-KEY (Pseudoscleroses, AND Cerebral) OR TITLE-ABS-KEY (Pseudosclerosis, AND Cerebral) OR TITLE-ABS-KEY (Hepatolenticular AND Degeneration AND Syndrome) OR TITLE-ABS-KEY (Degeneration AND Syndrome, AND Hepatolenticular) OR TITLE-ABS-KEY (Degeneration AND Syndromes, AND Hepatolenticular) OR TITLE-ABS-KEY (Hepatolenticular AND Degeneration AND Syndromes) OR TITLE-ABS-KEY (Syndrome, AND Hepatolenticular AND Degeneration) OR TITLE-ABS-KEY (Syndromes, AND Hepatolenticular AND Degeneration) OR TITLE-ABS-KEY (Hepato-Neurologic AND Wilson AND Disease) OR TITLE-ABS-KEY (Diseases, AND Hepato-Neurologic AND Wilson) OR TITLE-ABS-KEY (Hepato AND Neurologic AND Wilson AND Disease) OR TITLE-ABS-KEY (Hepato-Neurologic AND Wilson AND Diseases) OR TITLE-ABS-KEY (Wilson AND Disease, AND Hepato-Neurologic) OR TITLE-ABS-KEY (Wilson AND Diseases, AND Hepato-Neurologic) OR TITLE-ABS-KEY (Hepatocerebral AND Degeneration) OR TITLE-ABS-KEY (Degeneration, AND Hepatocerebral) OR TITLE-ABS-KEY (Degenerations, AND Hepatocerebral) OR TITLE-ABS-KEY (Hepatocerebral AND Degenerations) OR TITLE-ABS-KEY (Kinnier-Wilson AND Disease) OR TITLE-ABS-KEY (Diseases, AND Kinnier-Wilson) OR TITLE-ABS-KEY (Kinnier AND Wilson AND Disease) OR TITLE-ABS-KEY (Kinnier-Wilson AND Diseases) OR TITLE-ABS-KEY (Westphal-Strumpell AND Syndrome) OR TITLE-ABS-KEY (Westphal AND Strumpell AND Syndrome) OR TITLE-ABS-KEY (Westphal-Strumpell AND Syndromes) OR TITLE-ABS-KEY (Copper AND Storage AND Disease) OR TITLE-ABS-KEY (Copper AND Storage AND Diseases) OR TITLE-ABS-KEY (Disease, AND Copper AND Storage) OR TITLE-ABS-KEY (Diseases, AND Copper AND Storage) OR TITLE-ABS-KEY (Storage AND Disease, AND Copper) OR TITLE-ABS-KEY (Storage AND Diseases, AND Copper) OR TITLE-ABS-KEY (Progressive AND Lenticular AND Degeneration) OR TITLE-ABS-KEY (Degeneration, AND Progressive AND Lenticular) OR TITLE-ABS-KEY (Lenticular AND Degeneration, AND Progressive) OR TITLE-ABS-KEY (Neurohepatic AND Degeneration) OR TITLE-ABS-KEY (Degeneration, AND Neurohepatic) OR TITLE-ABS-KEY (Degenerations, AND Neurohepatic) OR TITLE-ABS-KEY (Neurohepatic AND Degenerations) OR TITLE-ABS-KEY (Hepatic AND Form AND of AND Wilson AND Disease) OR TITLE-ABS-KEY (Wilson AND Disease, AND Hepatic AND Form) AND LANGUAGE ( english )) AND PUBYEAR > 2000 AND PUBYEAR < 2021 AND ( LIMIT-TO ( DOCTYPE,"ar" ) OR LIMIT-TO ( DOCTYPE,"re" ) OR LIMIT-TO ( DOCTYPE,"le" ) OR LIMIT-TO ( DOCTYPE,"sh" ) )
